# Potential Role of Circulating Endoglin in Hypertension via the Upregulated Expression of BMP4

**DOI:** 10.3390/cells9040988

**Published:** 2020-04-16

**Authors:** Eunate Gallardo-Vara, Luis Gamella-Pozuelo, Lucía Perez-Roque, José L. Bartha, Irene Garcia-Palmero, J. Ignacio Casal, José M. López-Novoa, Miguel Pericacho, Carmelo Bernabeu

**Affiliations:** 1 Centro de Investigaciones Biológicas Margarita Salas, Consejo Superior de Investigaciones Científicas (CSIC), 28040 Madrid, Spain; eunate.gallardo@yale.edu (E.G.-V.); luisgamellap@gmail.com (L.G.-P.); irenegarciapalmero@gmail.com (I.G.-P.); icasal@cib.csic.es (J.I.C.); 2 Centro de Investigación Biomédica en Red de Enfermedades Raras (CIBERER), 28040 Madrid, Spain; 3 Yale Cardiovascular Research Center, Section of Cardiovascular Medicine, Department of Internal Medicine, Yale University School of Medicine, New Haven, CT 06511, USA; 4 Biomedical Research Institute of Salamanca (IBSAL) and Renal and Cardiovascular Physiopathology Unit, Department of Physiology and Pharmacology, University of Salamanca, 37007 Salamanca, Spain; luciap@usal.es (L.P.-R.); jmlnovoa@usal.es (J.M.L.-N.); 5 Division of Obstetrics and Maternal and Fetal Medicine, University Hospital La Paz, 28046 Madrid, Spain; jose.bartha@uam.es

**Keywords:** hypertension, preeclampsia, HHT, endoglin, endothelial cells, TGF-β, BMP4

## Abstract

Endoglin is a membrane glycoprotein primarily expressed by the vascular endothelium and involved in cardiovascular diseases. Upon the proteolytic processing of the membrane-bound protein, a circulating form of endoglin (soluble endoglin, sEng) can be released, and high levels of sEng have been observed in several endothelial-related pathological conditions, where it appears to contribute to endothelial dysfunction. Preeclampsia is a multisystem disorder of high prevalence in pregnant women characterized by the onset of high blood pressure and associated with increased levels of sEng. Although a pathogenic role for sEng involving hypertension has been reported in several animal models of preeclampsia, the exact molecular mechanisms implicated remain to be identified. To search for sEng-induced mediators of hypertension, we analyzed the protein secretome of human endothelial cells in the presence of sEng. We found that sEng induces the expression of BMP4 in endothelial cells, as evidenced by their proteomic signature, gene transcript levels, and BMP4 promoter activity. A mouse model of preeclampsia with high sEng plasma levels (*sEng^+^*) showed increased transcript levels of BMP4 in lungs, stomach, and duodenum, and increased circulating levels of BMP4, compared to those of control animals. In addition, after crossing female wild type with male *sEng^+^* mice, hypertension appeared 18 days after mating, coinciding with the appearance of high plasma levels of BMP4. Also, serum levels of sEng and BMP4 were positively correlated in pregnant women with and without preeclampsia. Interestingly, sEng-induced arterial pressure elevation in *sEng^+^* mice was abolished in the presence of the BMP4 inhibitor noggin, suggesting that BMP4 is a downstream mediator of sEng. These results provide a better understanding on the role of sEng in the physiopathology of preeclampsia and other cardiovascular diseases, where sEng levels are increased.

## 1. Introduction

The transforming growth factor-β (TGF-β) signaling system is well-established and includes soluble ligands, membrane receptors, and downstream Smad mediators, involved in the complex regulation of a plethora of biological processes that, among others, impact cardiovascular diseases [1,2,3,4,5]. Endoglin is a membrane co-receptor of the TGF-β family that is predominantly expressed by endothelial cells and is involved in vascular development, homeostasis, repair, and disease [6,7,8]. Thus, mutations in the human ENDOGLIN gene (ENG) cause Hereditary Hemorrhagic Telangiectasia (HHT) type 1, a dominant vascular disease that present with nose and gastrointestinal bleedings, telangiectases on skin and mucosa, and arteriovenous malformations in lung, liver, and brain [8,9,10]. Also, Eng-KO mice die in utero due to vasculogenic defects, suggesting a key role of endoglin in the vascular system [11].

Endoglin is a 180-kDa homodimeric transmembrane protein that contains a large extracellular region (561 amino acids) and a short (47 amino acids) cytosolic domain [12,13]. The juxtamembrane region of the endoglin ectodomain can be proteolytically targeted by the matrix metalloprotease 14 (MMP14; MT1-MMP) or by MMP-12 to release a soluble protein (either alone or in complex with exosomes), named sEng which encompasses most of its extracellular region [14,15,16,17,18,19]. Analysis of the three-dimensional structure of the endoglin ectodomain, has revealed the presence of an N-terminal orphan region (OR), and a C-terminal bipartite zona pellucida (ZP) module [20,21]. The OR of endoglin binds with high affinity to members of the TGF-β family, namely bone morphogenetic protein 9 (BMP9) and BMP10 [21,22,23]. Interestingly, rather than being an inhibitory ligand trap, the sEng/BMP9 complex is able to signal via membrane-bound endoglin in endothelial cells [24]. Noteworthy, dysregulated BMP signaling has been linked to vascular diseases, including HHT, pulmonary hypertension, and atherosclerosis, likely through endothelial dysfunction [25]. In addition to the well-recognized role of endoglin as a functional co-receptor of the TGF-β family ligands [26,27,28,29], its extracellular region can specifically interact with the TGF-β type I receptor ALK5 and with the TGF-β type II receptor [30,31]; it can also concurrently bind to BMP9 and the TGF-β type I receptor ALK1 in endothelial cells [21,22,31]. The ZP module of endoglin is predicted to be involved in polymerization with extracellular proteins, as for other ZP protein family members [21,32]. Moreover, the endoglin ZP module encompasses an accessible arginine-glycine-aspartic acid (RGD) sequence, which is a consensus binding motif for integrin recognition [12,21]. In this regard, the RGD motif of endoglin, in mature and precursor endothelial cells, appears to be actively involved in integrin-mediated cell adhesion through, at least, α5β1 and αvβ3 integrin family members [33,34,35,36].

The circulating sEng can be shed from membrane-bound endoglin [7,37,38] upon activation by endothelial injury, inflammation, or tumor necrosis factor α (TNF-α) stimuli [16,19,39,40]. Abnormally elevated levels of sEng have been found in several vascular-related pathologies [6,37,38], including preeclampsia, a multisystem disorder of high prevalence in pregnant women marked by the onset of hypertension, proteinuria or systemic endothelial dysfunction. If left untreated, preeclampsia can lead to serious, even fatal, complications for both mother and baby [41,42]. Noteworthy, several lines of evidence support a pathogenic role of sEng in cardiovascular conditions and diseases, including hypertension, endothelial dysfunction, anti-angiogenic activity, increased vascular permeability, vascular remodeling, and inflammation-associated leukocyte adhesion and transmigration [14,15,33,34,42,43,44,45].

Despite the emerging role that sEng plays in cardiovascular pathophysiology, its exact molecular mechanism of action remains elusive. In this study, we have sought to identify possible mediators of sEng activity. We show that sEng induces the expression of BMP4 in vitro and in vivo, and that sEng-induced arterial pressure elevation in mice overexpressing sEng is abolished in the presence of the BMP4 inhibitor noggin, suggesting that BMP4 is a downstream mediator of sEng. Taken together, this study reveals a novel avenue on the pathobiology of preeclampsia and other cardiovascular diseases, where sEng levels are increased.

## 2. Materials and Methods

### 2.1. Cell Culture

Human umbilical vein endothelial cells (HUVECs) were purchased from Lonza and used at early passages (3–5). HUVECs were grown on 0.2% gelatin (Sigma-Aldrich) pre-coated plates in endothelial basal medium (EBM2) supplemented with EGM2 SingleQuots (EBM2/EGM2 medium; Lonza). The human embryonic kidney cell line HEK293T was cultured in Dulbecco’s modified Eagle’s medium (DMEM, Gibco). Unless otherwise noted, cell media were supplemented with 10% heat-inactivated fetal bovine serum (FBS, Gibco), 2 mM L-glutamine, 100 U/mL penicillin, and 100 µg/mL streptomycin (Gibco). For sEng-induced BMP4 protein and mRNA expression assays, HUVECs monolayers were treated with different sEng concentrations (40 ng/mL or 100 ng/mL) for 24 h in serum-free EBM2/EGM2 medium with antibiotics. Then, cells were analyzed for BMP4 transcript levels, whereas culture supernatants were assayed for BMP4 protein levels by ELISA. All cell types were incubated at 37 °C in a humidified atmosphere with 5% CO_2_.

### 2.2. Protein Quantification of the Endothelial Secretome Using Isobaric Labeling (iTraq)

HUVECs were cultured in complete medium (EBM2/EGM2 with 10% FBS and antibiotics) to 80% confluence. Then, cells were incubated overnight in EBM2 medium with 1% FBS, followed by 24 h with serum-free EBM2 medium with or without 100 ng/mL of recombinant human sEng (Glu26-Gly586; 1097-EN, R&D Systems). After treatment, the medium was collected, and proteins were precipitated with acetone overnight at −20 °C. Quantitative proteomic analysis of the samples was carried out using the iTRAQ (Isobaric Tags for Relative and Absolute Quantification) labeling strategy as described [46,47,48]. Briefly, protein samples (50 μg each) from untreated (samples C1 and C2) and sEng-treated (samples S1 and S2) HUVECs were concentrated by high speed centrifugation for 20 min at 4 °C and digested with trypsin at 37 °C overnight with gentle stirring. The resulting peptides of the four samples (C1, S1, C2, and S2) were covalently labeled individually with the 4 isobaric reagents of the commercial kit (# 114, # 115, # 116, and # 117, respectively) (Supplementary Figure S1). The contents of the reaction tubes were pooled, desalted, and purified by ion exchange chromatography using Oasis MCX cartridges (Waters) to remove possible impurities. The resulting sample was dried under vacuum using the SpeedVac concentrator, at 30 °C for ~1 h. Subsequently, tryptic peptides were resuspended in an ampholyte solution (5% glycerol and 1% Ampholine^®^ [Sigma] in water), and fractionated on an OFFGEL Fractionator (Agilent Technologies) based on their isoelectric point using isoelectric focusing strips of 6 wells (Immobiline Dry Strip, pH 3–10, 13 cm; GE Healthcare). Eluted fractions were desalted using OMIX columns containing a C18 reverse phase resin (Millipore) and dried under vacuum in the SpeedVac. Subsequently, each sample was reconstituted in 5 μL of 0.1% formic acid and 2% acetonitrile (ACN) for mass spectrometry (MS) analysis. For protein identification and quantification, tryptic peptides were analyzed by Liquid Chromatography and Tandem Mass Spectrometry (LC-MS/MS) on the LTQ-Orbitrap Velos ion trap mass spectrometer (Thermo Scientific). The results obtained from the mass spectrometer (MS/MS peaks of each peptide) were analyzed, quantified, and identified by comparison with standardized human databases using the Mascot search engine (version 2.3, Matrix Science) with the Proteome Discoverer software (version 1.4.0.288; Thermo Scientific) and human Uniprot database. Of the 1,301 proteins identified in the secretome, 730 were quantified. Then, a list of 154 up-regulated (fold-induction > 1.05; Supplementary Table S1) or 122 down-regulated (fold-induction < 0.95; Supplementary Table S2) proteins were identified by comparing the secretome of sEng-treated HUVECs versus control samples. Among the dysregulated proteins, a stringent selection was applied by: (i) discarding proteins related to the proteasome because they are considered to be common contaminants; (ii) imposing a ratio (sEng-treated versus untreated) of > 1.24 or < 0.94 as a threshold; (iii) including only those proteins whose fold-change followed a similar trend in both replicates; and (iv) discarding those proteins that were identified with a single unique peptide, or showing a variability higher than 50%. In addition, only those proteins with a *p*-value ≤ 0.005 were considered significantly dysregulated.

### 2.3. RNA Expression Analysis by Quantitative Real-Time Polymerase Chain Reaction (qRT-PCR)

Total RNA from cultured HUVECs or previously homogenized mouse tissues was isolated and purified using the SpeedTools kit (Biotools), or the RNeasy kit (170-8891; iScript cDNA Synthesis kit; BioRad). One μg of total RNA from each sample was retrotranscribed into cDNA with the iScript cDNA Synthesis Kit (BioRad) in a final volume of 20 μL, following the manufacturer’s instructions. The resulting cDNA was used as a template for subsequent quantitative real-time PCR. For qRT-PCR assays of human BMP4, specific oligonucleotides labeled with FAM (Hs03676628_s1; TaqMan Gene Expression Assays, Applied Biosystems), and Roche’s FastStart Essential DNA Probes Master Mix containing Taq DNA Polymerase (Life Science). Amplification experiments were performed with the iQ5 thermal cycler (Bio-Rad). The qRT-PCR of murine Bmp4 was carried out using the iQTM SYBR^®^ Green Supermix (170-8880; BioRad), and mouse Bmp4 specific oligonucleotides (Forward, CGTTACCTCAAGGGAGTGGA; Reverse, ATGCTTGGGACTACGTTTGG). DNA amplification was performed with the Roche LightCycler 96 thermal cycler, using human or murine 18S ribosomal RNA as an internal control. Samples were analyzed in triplicate, and each experiment was repeated at least three times. Results were normalized with respect to the expression levels of the 18S ribosomal RNA by the 2^−ΔΔCt^ method.

### 2.4. ELISA of BMP4, sEng, and sFlts1

Human BMP4 from HUVECs culture supernatants or from human sera was measured with the Human BMP4 Quantikine ELISA Kit (DBP400; R&D Systems). To measure BMP4 in mouse plasma the Mouse BMP4 ELISA Kit (LSF13543; sensitivity range 15.6–1000 pg/mL; LSBio) was used. Concentration of human sEng in human or mouse sera was determined by ELISA (DNDG00; sensitivity range 0.2–10 ng/mL; R&D Systems), whereas concentration of sFlt1 in human sera was measured using an electrochemiluminescence immunoassay (Elecsys^®^ sFlt-1, Roche). All immunoassays were performed following the manufacturer’s instructions and measured in a GloMax multidetection system (Promega).

### 2.5. Plasmids, Cell Transfections and Reporter Assays

Transient transfections of HEK293T cells were carried out using Lipofectamin 2000 (Invitrogen), according to the manufacturer’s instructions. To measure the BMP4 gene promoter activity, the pEZX-PG04.1 commercial reporter vector (HPRM38607-PG04; GeneCopeia, Rockville, MD, USA) was used. This vector encodes the Gaussia luciferase driven by the human BMP4 promoter. Cell transfection with the pEZX-PG04.1 vector in the presence or absence of sEng treatment was performed, as indicated. After forty-eight hours, cell lysates were analyzed using dual-luciferase reporter assay system (Promega) in a GloMax multi-detection system luminometer (Promega). Transfection efficiency was normalized to Renilla luciferase activity.

### 2.6. Human Blood Samples

In total, 34 women allocated into two groups, 16 with preeclampsia and 18 controls, participated in the study. All of them signed an informed consent form. The study was approved by the Local Ethical Committee. Maternal blood (5 mL) was collected in fasting state, allowed to clot and centrifuged for 10 min. All serum samples were frozen at −80 °C until the day of the analysis. Preeclampsia was defined as the presence of pregnancy-induced hypertension (maternal blood pressure > 140/90 mmHg) emerging for the first time after 20 weeks of gestation plus proteinuria (≥300 mg in 24-h urine). Of the 16 women with preeclampsia, 5 showed early-onset preeclampsia (≤34 weeks’ gestation) while 11 had late-onset preeclampsia (>34 weeks’ gestation). Based on their symptoms, 11 women were classified as having severe preeclampsia and 5 had mild preeclampsia. Maternal age was similar in both groups (36.54 ± 4.99 vs. 34.52 ± 5.12 years) while gestational age was significantly lower in the preeclampsia group (34.31 ±3.80 vs. 39.10 ± 1.02 weeks) (*p* < 0.001).

### 2.7. Mice

All procedures were approved by the Committee for the Care and Use of Animals of the University of Salamanca and complied with the current guides of the European Union and the U.S. Department of Health and Human Services for the Care and Use of Laboratory Animals. Transgenic mice overexpressing human sEng (*sEng^+^*) on the CBAxC57BL/6J background were generated at the Genetically Modified Organisms Generation Unit (University of Salamanca, Spain) by microinjection in fertilized eggs of a pCAGGS vector containing a truncated endoglin construct (amino acids 26-437), as previously described [15]. Littermates who do not carry the transgene were used as control or wild type (WT) animals.

### 2.8. Mouse Blood and Tissue Collection

Mouse blood samples were taken from the jugular vein, using EDTA as anticoagulant. For the extraction of the different organs, animals were anesthetized with a sub-lethal dose of sodium pentobarbital. Then, a deep and extensive incision was made, of both skin and muscular layer through the *linea alba* of the abdomen, leaving the entire visceral mass accessible. Next, the thoracic cage was accessed, and the heart was cannulated through the apex. Through this route, a solution of isotonic saline (0.9% NaCl) with heparin (1:1000) was circulated systemically at 37 °C at a pressure of ~100 mmHg. The circulatory system was opened through the ascending vena cava section and organs were perfused, for 5–10 min. The lungs, stomach and first third of the small intestine (duodenum) were isolated, and then processed for immunohistochemistry (fixation) or qRT-PCR (freezing in liquid nitrogen at −80 °C) analyses of BMP4.

### 2.9. In Vivo Experiments with Osmotic Pumps

Treatments with noggin were carried out in hypertensive *sEng^+^* transgenic mice and control animals. Murine noggin (AF-250-38, Peprotech) was loaded in osmotic pumps (Alzet Osmotic Pump Mod. 2001, Alzet), which provide a constant flow of 1 µL/hour for 7 days. Control pumps were loaded with vehicle (physiological serum, 0.9% NaCl). Osmotic pumps were implanted subcutaneously and adjusted to release 1 µg of noggin/hour/kg of animal weight. On subsequent days post-implantation, blood pressure was measured, and blood samples were taken.

### 2.10. Mouse Model of Preeclampsia

Male transgenic *sEng^+^* mice were crossed with female wild type (WT) mice (CBAxC57BL/6J background). Pregnant WT female resulting from this cross were named as fWT(*sEng^+^*). Pregnant mice resulting from the cross between a male WT with female WT mice (CBAxC57BL/6J background), were named as fWT(WT). Blood samples were taken from the jugular vein and blood pressure was determined by the tail-cuff technique, as above. This model of preeclampsia displays a sEng-induced hypertensive effect in pregnant fWT(*sEng^+^*) [15,49].

### 2.11. Blood Pressure Measurements in Mice

Systolic blood pressure was determined by the tail-cuff plethysmography technique using a NIPREM 645 (Cibertec) device, after mice were accustomed to the procedure. Animals were trained daily for 1 week to get used to the system, before the final measurements. The measurements were always taken at the same time (between 9 and 12 a.m.), in a dark room isolated from noise and with a constant temperature, in order to avoid that both external and circadian factors could alter measurements. In the absence of this “training” process, mice experience some degree of anxiety and stress during balloon inflation on the tail, as evidenced by elevated heart rate. Although the tail-cuff technique has some limitations, it is a reliable method to assess the effect of a drug on arterial pressure or to compare the basal arterial pressure between two strains of mice [50]. In fact, reproducible measurements of arterial pressure with tail-cuff plethysmography in mice can be obtained when animals are “trained” on the procedure, with the purpose of reducing stress-related effects. Also, arterial pressures obtained non-simultaneously by radiotelemetry and tail-cuff show a good correlation [50].

### 2.12. Immunohistochemistry

One of the lungs, one half of the stomach, and one half of the intestine were fixed in 4% formaldehyde. For inclusion in paraffin, tissues were first subjected to a progressive dehydration at increasing concentrations of ethanol (from 50% to absolute ethanol) and subsequently in xylene. Once dehydrated, samples were incubated with paraffin at 60 °C for 24 h and allowed to solidify. Two-µm sections of paraffin blocks were dewaxed and rehydrated, first with xylene, then with decreasing concentrations of ethanol, and finally distilled water. For immunostaining, epitopes were unmasked by heat in a Tris-EDTA solution (10 mM Tris, 1 mM EDTA, pH 8). Subsequently, samples were incubated with an anti-BMP-4 rabbit monoclonal antibody (Clone # 1128D, MAB5020; R&D Systems) at a 1/100 dilution for 40 min, followed by a secondary antibody bound to horse radish peroxidase (Discovery ChromoMap DAB Kit 760-159, Roche). The presence of the antigen was visualized by a commercial kit using diaminobenzidine (DAB, Roche). Samples were counterstained with hematoxylin, dehydrated with ethanol and xylol, and assembled using the DPX mounting medium (Sigma). Photo documentation was carried out with an Olympus BX51 microscope at 20× magnification.

### 2.13. Statistical Analysis

Results are shown as mean ± SD and differences in mean values were analyzed using Student’s *t* test. For data obtained from human sera, the Graphpad Prism v.7 was used. Normality of raw data in each group was analyzed using Kolmogorov–Smirnova and Shapiro–Wilk statistical test. As both maternal sEng and BMP4 were distributed in a non-parametric manner, we used log-transformed values for correlations (Pearson’s correlation coefficient). Asterisks indicate statistically significant values between selected conditions (* *p* < 0.05; ** *p* < 0.01; *** *p* < 0.001; ns, not significant).

## 3. Results

### 3.1. Identification of sEng-Induced Downstream Mediators in Human Endothelial Cells

**Figure 1:**
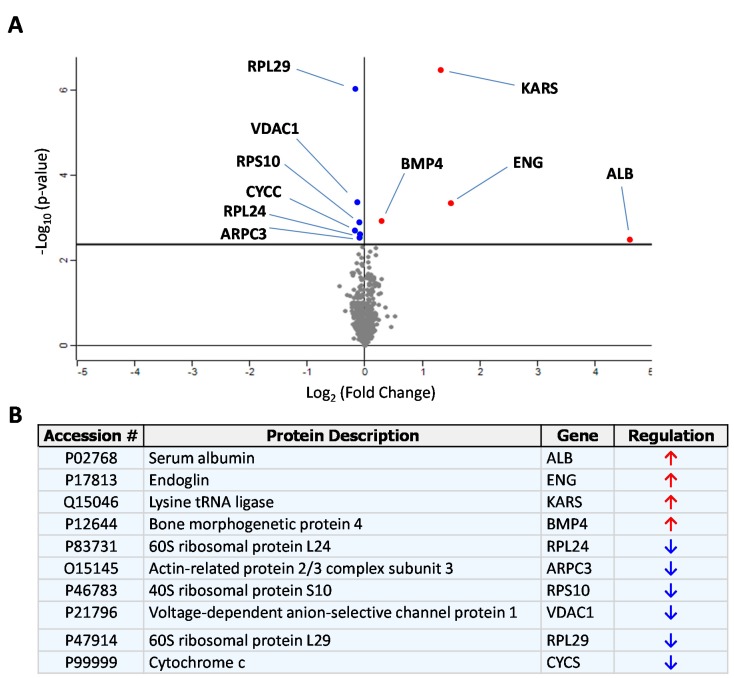
iTRAQ identification of differentially secreted proteins by human umbilical vein endothelial cells (HUVECs) in the presence of sEng. (**A**) Volcano type graph where secreted proteins from sEng-treated HUVECs (HUVECs-sEng) that change significantly with respect to the untreated control (HUVECs-Control) are indicated. The horizontal axis represents fold changes in induction of the ratio HUVECs-sEng/HUVECs-Control, considering negligible differences those values closer to 0. The vertical axis represents the -Log10 (p-value), and the continuous horizontal line plotted at the value of 2.3, is equivalent to a p-value of 0.005. The central gray point cloud represents quantified but not statistically significant proteins. Inhibited or over-expressed proteins with statistically significant differences after treatment with sEng are represented in blue (upper left quadrant) or red (upper right quadrant), respectively. (**B**) Table showing the list of proteins represented in the graph in which statistically significant differences were found, including the corresponding access number in the UniProt database (http://www.uniprot.org/uniprot/).

Recombinant sEng, encompassing the extracellular domain of human endoglin, was incubated with HUVECs monolayers in the presence of serum-free medium and quantitative proteomic analysis of the secretome was carried out using iTRAQ labeling, followed by tryptic digestion and mass spectrometry analysis. This approach allowed the identification of those proteins whose levels were altered in the presence of sEng. A preliminary selection identified 154 up-regulated and 122 down-regulated proteins when comparing the secretome of sEng-treated HUVECs versus control samples (Supplementary Tables S1 and S2, respectively). Additional stringent criteria (see Materials and Methods) led to the selection of only nine proteins (Figure 1). The volcano plot of Figure 1A shows the nine proteins identified, whose levels are increased (upper right quadrant) or decreased (upper left quadrant) after treatment with sEng. The names of each protein are indicated in the table of Figure 1B. The most upregulated proteins were endoglin and albumin, as expected from the fact that cells were treated with exogenous sEng containing bovine albumin as a carrier, and both human and bovine albumin share an identity of 77% in their sequences. Upon sEng treatment, the increased endogenous proteins were lysyl-tRNA synthetase (KARS) and bone morphogenetic protein 4 (BMP4). By contrast, the levels of 60S ribosomal protein L24 (RLP24), actin-related protein 2/3 complex subunit 3 (ARPC3), 40S ribosomal protein S10 (RPS10), voltage-dependent anion-selective channel protein 1 (VDAC1), and cytochrome c (CYCS) were found to the decreased upon sEng treatment. Of note, gene ontology analyses revealed that theses endogenous proteins were located in different subcellular compartments, including the cytoplasm, nucleus, membrane, and extracellular subsets. Among all these proteins, BMP4 was selected for further studies based on gene ontology studies on subcellular location, molecular function and biological processes. In fact, BMP4 is a ligand of the TGF-β signaling pathway, with endoglin acting as an auxiliary receptor, and a soluble factor that targets the vasculature, where endoglin plays a key functional role [3,6,51,52,53].

### 3.2. Expression of BMP4 is Induced by sEng in Human Endothelial Cells In Vitro

**Figure 2:**
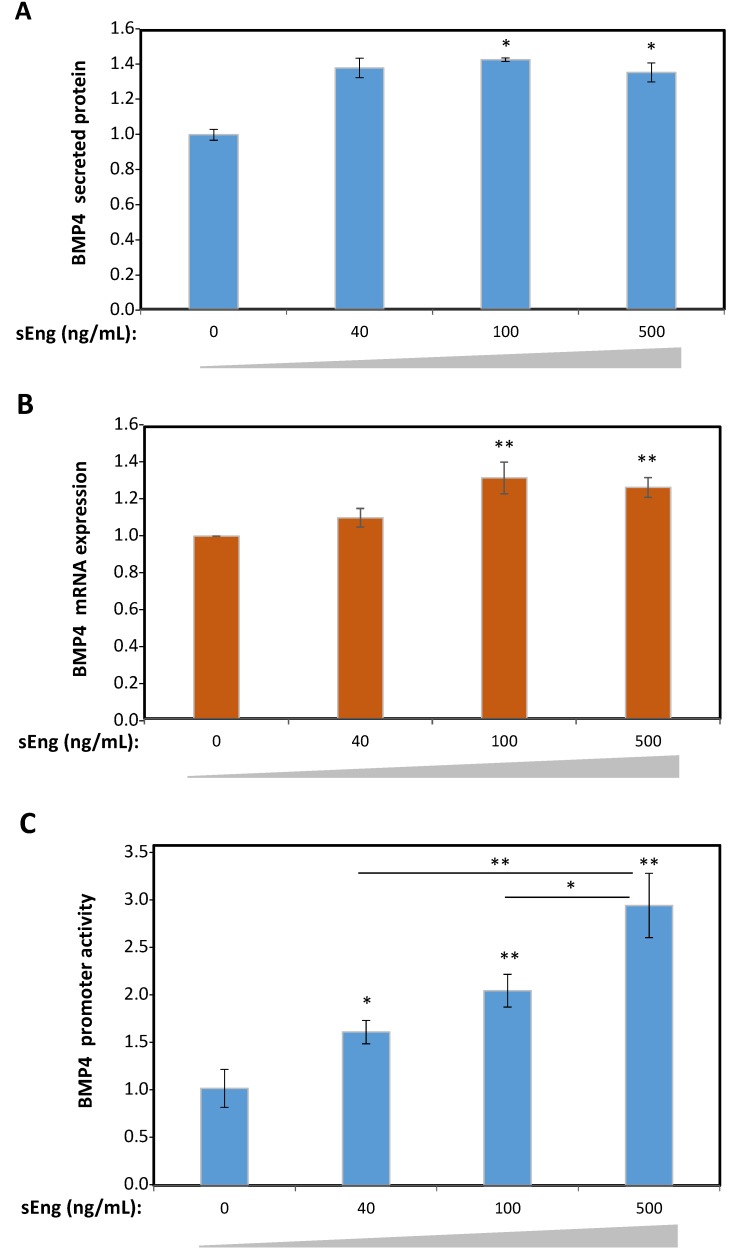
BMP4 expression upon in vitro treatment of cells with sEng. (**A**,**B**) HUVECs were treated with sEng at the indicated concentrations. Protein levels of BMP4 secreted into the medium were determined by ELISA and normalized to control (**A**). Transcript levels of BMP4 were measured by qRT-PCR and values normalized to the control condition (**B**). (**C**) sEng transactivates the BMP4 promoter. HEK293T cells were transiently transfected with the pEZX-PG04.1 vector encoding the Gaussia luciferase driven by the human BMP4 promoter. Cell transfections were performed in the presence of increasing concentrations of sEng and after forty-eight hours, the luciferase activity of cell lysates was measured by luminometry. Unless otherwise indicated, p values are referred to the control condition. (* *p* < 0.05; ** *p* < 0.01).

To validate the sEng-dependent upregulation of BMP4 observed in the proteomic data, protein and transcript expression studies were carried out in HUVECs. Cells treated with increasing concentrations of sEng showed significantly increased levels of BMP4 secreted into the culture medium compared to untreated HUVECs, as evidenced by ELISA (Figure 2A). Parallel qRT-PCR experiments demonstrated that mRNA levels of BMP4 were also increased in HUVECs upon sEng treatment (Figure 2B). These results suggest that sEng was involved in the regulated expression of BMP4 at the transcriptional level. To test this hypothesis, a luciferase-based reporter vector where expression of the luciferase gene is driven by the human BMP4 promoter, was used to transfect HEK293T cells. As shown in Figure 2C, a concentration-dependent activation of the BMP4 promoter construct was observed upon treatment with sEng. Taken together, the above results demonstrate that sEng stimulates the cellular expression of BMP4 in vitro.

### 3.3. Levels of sEng and BMP4 Correlate with Each Other in Sera from Pregnant Women

**Figure 3:**
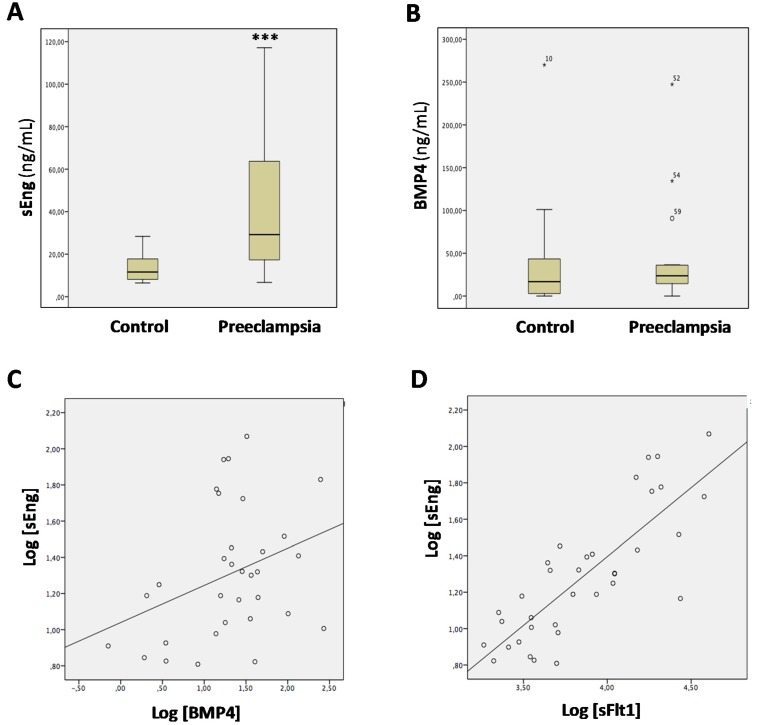
Correlation between sEng and BMP4 levels in sera from pregnant women. Sera from pregnant women without (control; *n* = 19) or with preeclampsia (*n* = 16) were analyzed by ELISA to determine protein levels of sEng (**A**; *** *p* = 0.001), BMP4 (**B**; *p* = 0.13) and sFlt1. In panel **B**, the presence of high-outliers (*^,^°) is indicated. Correlation analysis between sEng and BMP4 levels (**C**; *r* = 0.35, *p* = 0.03), and between sEng and sFlt1 levels (**D**; *r* = 0.81, *p* < 0.0001) are shown.

Circulating levels of sEng are elevated in preeclampsia, a life-threatening condition in some pregnant women [37,54,55]. However, little is known about BMP4 levels in preeclamptic women. Thus, we analyzed the possible correlation between sEng and BMP4 levels in a cohort of pregnant women. As expected, women with preeclampsia presented statistically significant higher levels of sEng than pregnant healthy women (Figure 3A; 29.21 [range 15.98–65.67] versus 11.60 [range 8.07–18.54], respectively; *p* = 0.001). Serum levels of BMP4 were also higher in women with preeclampsia than in controls, but this trend did not reach statistical significance (Figure 3B; 23.66 [range 14.26–36.27] versus 16.78 [range 2.67–43.54], respectively, *p* = 0.13). Interestingly, a positive correlation between sEng and BMP4 levels was observed in the whole population of pregnant women (Figure 3C; *r* = 0.35, *p* = 0.03). In addition, a positive correlation between sEng and sFlt1 was also observed (Figure 3D; *r* = 0.81, *p* < 0.0001), in agreement with previous reports [56,57,58]. These results support the hypothesis that increased serum levels of sEng may underlie the upregulated expression of BMP4 in vivo.

### 3.4. Expression of BMP4 is Enhanced by sEng in a Mouse Model

**Figure 4:**
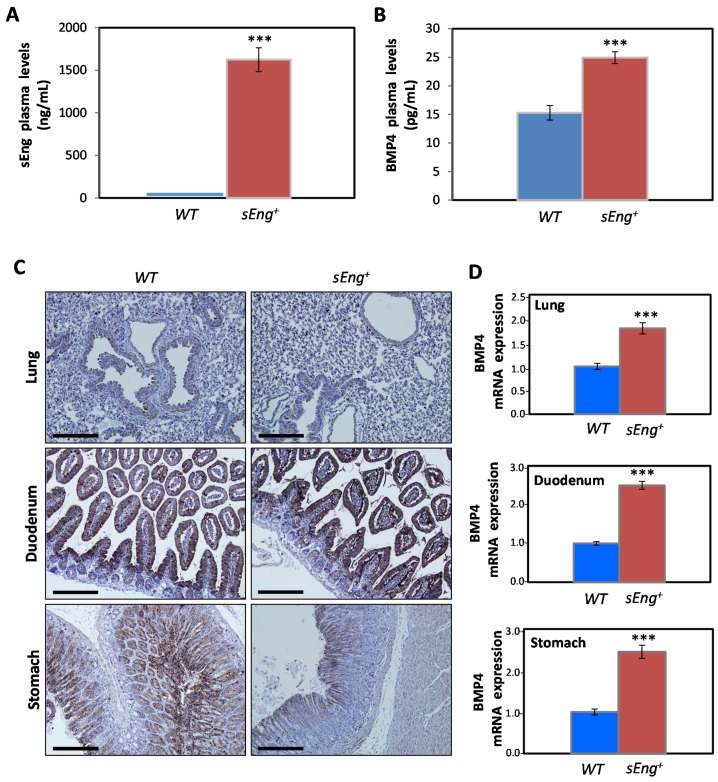
Expression of BMP4 in *sEng^+^* transgenic mice. (**A**,**B**) Plasma levels of BMP4 and sEng. (**A**) Phenotyping of mice used in the study. Plasma levels of sEng present in WT (*n* = 35) and *sEng^+^* (*n* = 35) animals were determined by ELISA. (**B**) Plasma levels of BMP4 in WT (*n* = 6) and *sEng^+^* (*n* = 5) mice, as measured by ELISA. The mean and the standard error of the mean are represented. (*** *p* < 0.001). (**C**) Immunohistochemical staining of BMP4 in mouse tissues. Lung, duodenum and stomach sections from WT or *sEng^+^* mice were stained with anti-BMP4 (brown color) and counterstained hematoxylin (blue color), as described in Materials and Methods. The images were taken at ×20 magnification. Scale bars, 50 µm. (**D**) Gene expression levels of BMP4 in mouse tissues. Quantification by qRT-PCR of BMP4 mRNA expression in lung (*n* = 7 animals for each genotype), duodenum (*n* = 7 WT mice and *n* = 8 *sEng^+^* mice), stomach (*n* = 5 WT mice and *n* = 7 *sEng^+^* mice) from WT or *sEng^+^* mice. Results were normalized, using 18R ribosomal RNA expression as internal control. The mean and the standard error are represented. (*** *p* < 0.001).

Next, we assessed whether the sEng-induced expression of BMP4 also occurred in vivo. To this end, a transgenic mouse line overexpressing human sEng (*sEng^+^*) was used [15]. Because these transgenic animals show variable levels of recombinant sEng in plasma, a minimum threshold of 1000 ng/mL of plasma sEng was established for animals to be included in the study group. The average plasma levels of human sEng in the test animals were around 1600 ng/mL, while control littermate mice showed negligible amounts of recombinant sEng (Figure 4A). In a subset of these *sEng^+^* mice, plasma levels of BMP4 were around 25 ng/mL, compared to 15 ng/mL observed in control animals (Figure 4B), and these differences showed a strong statistical significance.

In order to delve deeper into the source of the circulating BMP4, several tissues were subjected to immunostaining with anti-BMP4 antibodies (Figure 4C). It is noteworthy that although BMP4 tissue expression has been described in humans [59], similar studies in mouse tissue are scarce. Among the organs selected for this study, lungs were included because: (i) they express prominent levels of BMP4, and (ii) the endothelium of this highly vascularized organ may serve to corroborate the findings obtained in vitro with HUVECs. In addition, stomach and duodenum were also selected as they show a high level of BMP4 expression in humans [59]. As shown in Figure 4C, the lung expression pattern of BMP4, in both WT and *sEng^+^* mice, was located in the basal lamina of the bronchial tree, as well as in the wall and endothelium of blood vessels and in pulmonary macrophages. In the duodenum, the overall intensity of the labeling observed was much higher than that in the lung. Most of the BMP4 staining was located in cells of the intestinal epithelium, being more evident towards the apical zone of the cells, perhaps due to the characteristic basal location of the nuclei in this cell type. The remaining areas of the intestinal wall showed a weaker staining compared to the intestinal epithelium. In the case of the stomach, the BMP4 staining was less intense than that of the duodenum and appears to be mainly located in the cells that line the apical part of the mucosa, and to a lesser degree in the lamina muscularis mucosae (Figure 4C). While BMP4 was readily detected in these organs by IHC, due to the qualitative nature of this technique and the fact that BMP4 is released into circulation, no quantitative differences in BMP4 expression could be observed between WT and *sEng^+^* mouse tissues. To assess whether sEng was able to modify the gene expression of BMP4 in the different organs, qRT-PCR experiments were carried out (Figure 4D). The expression of BMP4 was clearly upregulated in the lung (~1.8-fold induction), duodenum (~2.5-fold induction), and stomach (~2.5-fold induction) of *sEng^+^* compared to WT mice and these differences were highly significant. Taken together, the above results suggest that the increased plasma levels of BMP4 are derived from the upregulated BMP4 gene expression induced by sEng in different organs.

### 3.5. BMP4 Is a Mediator of the sEng-Dependent Hypertensive Effect in Mice

**Figure 5:**
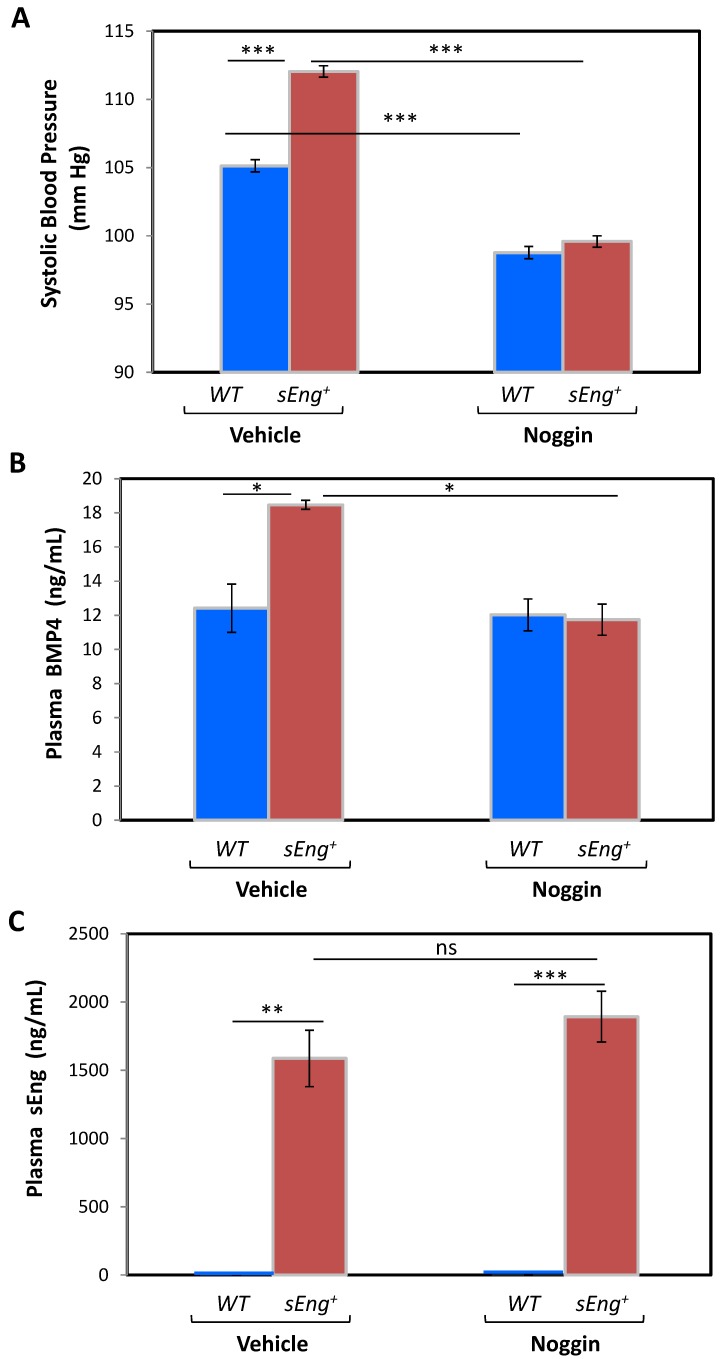
Effect of noggin administration on systolic blood pressure. Pumps loaded with either vehicle or noggin were implanted in WT or *sEng^+^* mice and four days later, arterial pressure (**A**) as well as plasma BMP4 (**B**) and sEng (**C**) levels were measured. *n* = 4 in each noggin-treated group; *n* = 3 in each vehicle-treated group. (* *p* < 0.05; ** *p* < 0.01; *** *p* < 0.001).

The sEng-induced upregulated expression of BMP4 prompted us to postulate that BMP4 could be a mediator of the biological function of sEng *in vivo*. In fact, both BMP4 [60,61] and sEng [15,37] appear to play an active role in the abnormal increase in arterial pressure. Therefore, we designed an experiment by inhibiting BMP4 in *sEng^+^* mice, which show increased arterial pressure [15]. Inhibition of BMP4 was achieved with noggin, a 64-kDa homodimeric glycoprotein, known to be an inhibitor of BMPs, in particular by binding and sequestering BMP4 [62,63,64]. Osmotic pumps with noggin or vehicle were implanted in mice of both genotypes (WT and *sEng^+^*), and animals were accustomed to tail blood pressure (BP) measurements for several days. Among animals implanted with vehicle-loaded osmotic pumps, *sEng^+^* mice displayed a statistically significant higher systolic BP (SBP) compared to WT mice at day 4 (Figure 5), in agreement with previous studies [15]. Noteworthy, upon noggin treatment a significant reduction in SBP values was observed in both genotypes, compared to vehicle-treated animals; the decrease in *sEng^+^* mice being much greater than that in WT (Figure 5A). As a result of the noggin treatment, no significant differences in the final SBP were found between both genotypes. Similar results to those of day 4 were also observed at day 7 post-implantation (data not shown). Concomitant plasma measurements revealed that upon noggin treatment in *sEng^+^* mice, BMP4 levels were similar to the levels observed in WT animals, further supporting the BMP4 involvement in SBP changes (Figure 5B). Moreover, sEng plasma levels were not significantly affected by the presence of noggin in *sEng^+^* mice (Figure 5C).

**Figure 6:**
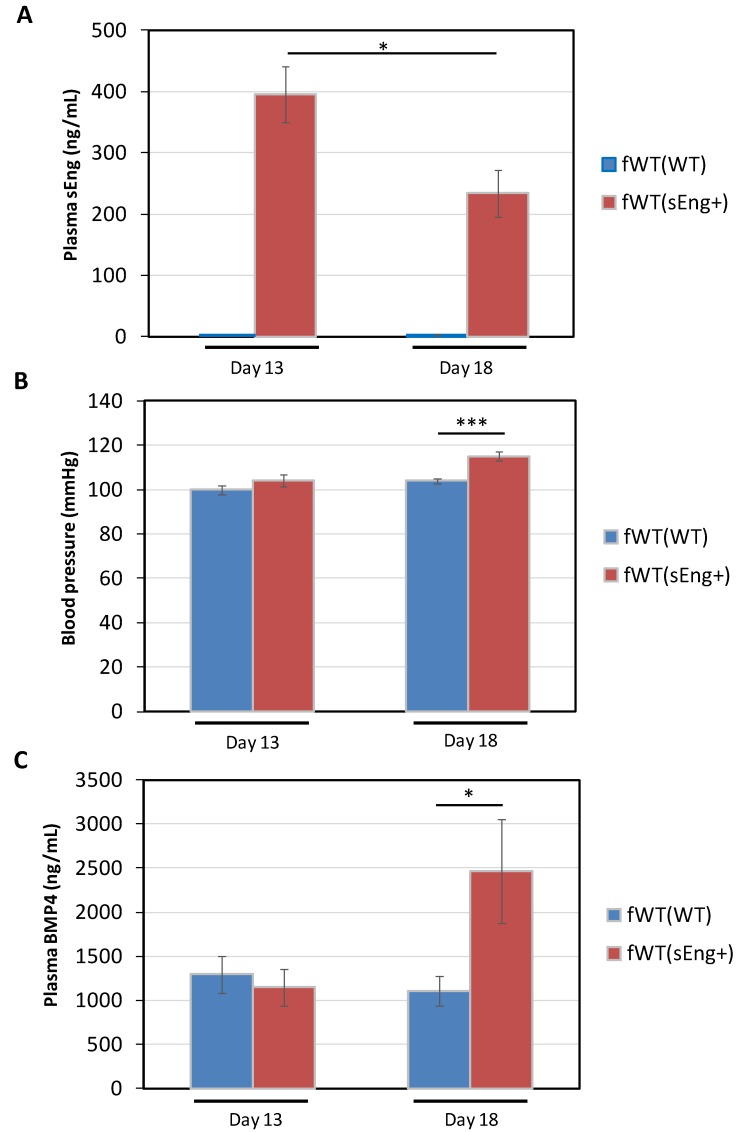
Analysis of plasma sEng and BMP4 levels and blood pressure in a model of preeclampsia. Wild type (WT) female mice were crossed with either *sEng^+^* or WT males. The resulting WT pregnant females bearing *sEng^+^* fetuses [fWT(*sEng^+^*)] or WT fetuses [fWT(WT)] were analyzed for sEng (**A**) and BMP4 (**C**) plasma levels, as well as SBP (**B**) at days 13 and 18 after pregnancy. (**A**,**C**) *n* = 9 mice for each group. (**B**) *n* = 14 mice for each group. (* *p* < 0.05; *** *p* < 0.001).

Further support for the involvement of BMP4 in the hypertensive effect was obtained by crossing *sEng^+^* males with WT females. The resulting WT pregnant female bearing *sEng^+^* fetuses [fWT(*sEng^+^*)] constitutes a useful model of preeclampsia, where the kinetic changes in sEng and BMP4 plasma levels as well as in SBP, all of them related to placental development, can be monitored [15,49]. Pregnant WT females bearing WT fetuses [fWT(WT)] were used as controls. After 13 days of pregnancy, fWT(*sEng^+^*) mice exhibited significantly higher plasma levels of sEng than pregnant fWT(WT) females, in which these levels were almost undetectable. The levels of sEng in pregnant fWT(*sEng^+^*) females were slightly reduced at the end of pregnancy (day 18) (Figure 6A). Interestingly, despite the well-described hypertensive effect of sEng, at day 13 of pregnancy SBP was not significantly higher in fWT(*sEng^+^*) compared with fWT(WT) mice. However, at day 18 of pregnancy, SBP was significantly higher in fWT(*sEng^+^*) than in fWT(WT), suggesting the existence of a sEng-induced mediator that could account for the delay between increased sEng levels and augmented SBP (Figure 6B). Compatible with this hypothesis, we found that plasma levels of BMP4 were significantly higher in fWT(*sEng^+^*) than in fWT(WT) mice at day 18 of pregnancy, coinciding with the increased SBP found in pregnant fWT(*sEng^+^*) mice at this day (Figure 6C).

Taken together, these results suggest that BMP4 is a downstream target of sEng that mediates sEng-induced hypertensive effect.

## 4. Discussion

Proteomic analysis of the secretome from sEng-treated human endothelial cells, combined with gene expression analysis, led us to demonstrate that sEng induces transcript and protein levels of BMP4. This upregulation appears to involve the transcription machinery as sEng enhances the BMP4 gene promoter activity, suggesting a gene expression-related function for sEng. In this line, endoglin deficiency in HHT1 endothelial cells, or overexpression in human cells, can regulate the expression of a wide range of target genes at the transcript level [65,66,67]. Also, a number of potential novel interactors of sEng located in the nucleus have been recently identified [68]. Furthermore, sEng bound to BMP9 is able to intracellularly signal via membrane-bound endoglin in endothelial cells, rather than being an inhibitory ligand trap [24]. Whether the sEng-dependent regulation of BMP4 gene expression is mediated by the downstream TGF-β/BMP signaling pathways, a yet-to-discover function of sEng, or both, remains to be explored.

In agreement with the sEng-dependent upregulation of BMP4 observed in vitro, we found a positive correlation between the levels of both proteins in the sera of pregnant women with or without preeclampsia, a disease where sEng levels are abnormally elevated [37,54,55,56,69]. Even more, in transgenic mice that overexpress human sEng, we found that tissue transcript levels and plasma concentration of BMP4 were significantly higher than those of WT animals. The BMP4 upregulation was evident in lung, stomach, and duodenum, organs where BMP4 protein is readily detected by immunohistochemistry, suggesting that they are a potential source of circulating BMP4. In this context, it has been reported that BMP4 is expressed in the intravillus mesenchyme and is involved in epithelial cell renewal of the intestine [70,71]. Moreover, BMP4 appears to be at the molecular basis of certain lung diseases such as pulmonary arterial hypertension (PAH), chronic obstructive pulmonary disease (COPD) or hypoxic pulmonary hypertension [25,72,73,74,75].

**Figure 7:**
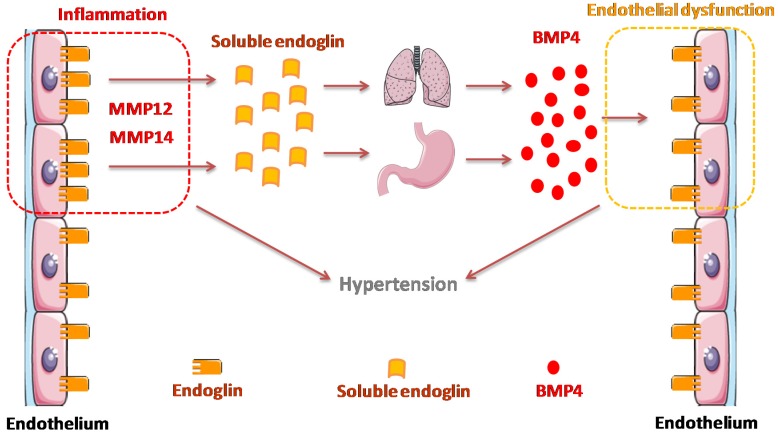
Hypothetical model for BMP4 in sEng-dependent effect in hypertension. Upon inflammation, MMP12 and/or MMP14 trigger the release of sEng in endothelial cells by proteolytically cleaving membrane-bound endoglin. In turn, sEng stimulates the expression of BMP4 in different organs, including lung, stomach and duodenum. Next, circulating BMP4 contributes to endothelial dysfunction, leading to hypertension.

Our data suggest that BMP4 is a downstream mediator of the sEng-induced hypertensive effect (Figure 7). Supporting this hypothesis, independent studies have shown that BMP4 and sEng have in common their involvement in hypertension, a hallmark of preeclampsia a disease associated with increased sEng levels. Thus, overexpression of sEng driven by adenovirus in rats or by transgenesis in mice induces hypertension [15,37]. In addition, sEng plasma levels are decreased in hypertensive patients upon anti-hypertensive treatment with an angiotensin converting enzyme (ACE) inhibitor [76]. Similar to sEng, BMP4 induces hypertension in mice [60]. Interestingly, endothelial dysfunction precedes and mediates the BMP4-induced hypertensive effect [60,61,77]. This is in agreement with the presence of a generalized endothelial dysfunction in preeclampsia likely due to placental factors such as sEng [41,42,58,69]; syncytiotrophoblasts being the major source of sEng [17,38]. In fact, it has been reported that sEng contributes to endothelial dysfunction. Thus, sEng synergizes with hypercholesterolemia to aggravate endothelial and vessel wall dysfunction in vivo [43,45] and shows pro-inflammatory activity via nuclear factor-kappa B (NFkB) and interleukin 6 (IL6) in human endothelial cells in vitro [78]. Furthermore, exosomes from preeclamptic women induced vascular dysfunction by delivering sEng to endothelial cells [18]. Because abnormal levels of sEng are found not only in preeclampsia, but also in several vascular- and inflammation-related pathological conditions [6,7,79,80,81,82,83], these results suggest that BMP4 may be a sEng-induced mediator, regulating endothelial function in these pathologies. Accordingly, BMP4 and BMP4 inhibitors like noggin could be considered as potential therapeutic targets in the above diseases. Further studies are needed to assess the regulated expression and function of BMP4 in those pathological conditions associated with elevated levels of sEng.

## 5. Conclusions

In summary, here we have identified BMP4 as a downstream mediator of sEng. Given the involvement of sEng in different pathological contexts, including preeclampsia, these findings open up a new research avenue to better understand its mechanism of action. Future independent studies remain to be performed in order to address the exact mechanism by which sEng enhances BMP4 expression, as well as the functional and pathophysiological significance of this newly discovered sEng/BMP4 association.

## Supplementary Materials

The following is available online at https://www.mdpi.com/2073-4409/9/4/988/s1, Figure S1: Scheme of the protein isobaric labeling of the HUVECs secretome; Table S1: Upregulated proteins upon sEng treatment; Table S2: Downregulated proteins upon sEng treatment.
