# Supplementary material for: Potential Role of Circulating Endoglin in Hypertension via the Upregulated Expression of BMP4
Source: Cells. 2020 Apr 16;9(4):988. doi: 10.3390/cells9040988 (PMC7226995; doi:10.3390/cells9040988)
Supplement: Supplementary file 1 [file cells-09-00988-s001.pdf]

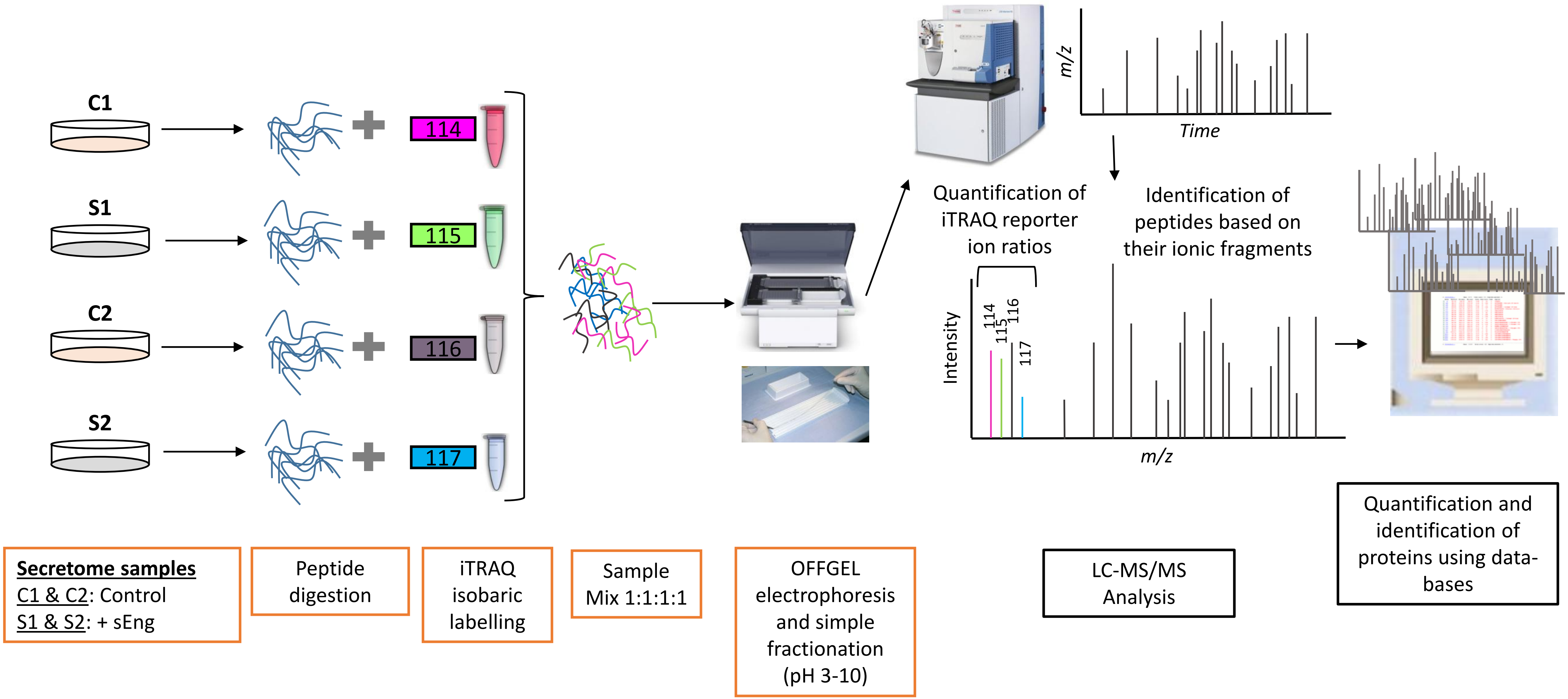

Figure S1. Scheme of the protein isobaric labeling of the HUVECs secretome. HUVECs were treated in EBM2/1% FBS medium with (S1, S2) or without (C1,C2) 100 ng/mL sEng for 24h. Each of the four samples (C1, S1, C2, S2) was labeled with a different isobaric reagent (#114, #115, #116 and #117, respectively), followed by fractionation, purification, peptide analysis by LC-MS/MS and protein identification and quantification.

Supplementary Table S1. Upregulated proteins upon sEng treatment (≥5%)*

Accession	Protein Name	Gene Name	ΣCoverage	ΣUnique peptides	ΣPSMs	solEng/Control Average	SD
P02768	Serum albumin	ALB	3.61	3	434	5.78	0.993
P17813	Endoglin	ENG	10.18	5	9	2.62	0.197
Q15046	Lysyl-tRNA synthetase	KARS	8.54	4	4	2.31	0.029
O43242	26S proteasome non-ATPase regulatory subunit 3	PSMD3	3.93	2	2	1.55	0.382
P08493	Matrix Gla protein	MGP	21.36	2	7	1.40	0.464
P63220	40S ribosomal protein S21	RPS21	28.92	2	7	1.39	0.522
P43251	Biotinidase	BTD	2.39	1	2	1.27	0.094
P15374	Ubiquitin carboxyl-terminal hydrolase isozyme L3	UCHL3	8.70	1	2	1.27	0.202
P51149	Ras-related protein Rab-7a	RAB7A	14.49	3	4	1.25	0.069
P12644	Bone morphogenetic protein 4	BMP4	5.15	1	1	1.24	0.043
P54136	Arginyl-tRNA synthetase. cytoplasmic	RARS	3.33	2	3	1.24	0.083
P53041	Serine/threonine-protein phosphatase 5	PPP5C	2.20	1	1	1.23	0.300
Q6P2Q9	Pre-mRNA-processing-splicing factor 8	PRPF8	3.38	5	8	1.23	0.149
O75475	PC4 and SFRS1-interacting protein	PSIP1	2.83	2	3	1.19	0.551
P61086	Ubiquitin-conjugating enzyme E2 K	UBE2K	16.50	2	6	1.19	0.037
Q8IZP2	Protein FAM10A4	ST13P4	5.42	1	2	1.18	0.105
P13489	Ribonuclease inhibitor	RNH1	6.51	2	4	1.18	0.101
P52926	High mobility group protein HMGI-C	HMGA2	17.43	1	2	1.18	0.091
P50552	Vasodilator-stimulated phosphoprotein	VASP	15.26	5	12	1.17	0.107
P0C0S5	Histone H2A.Z	H2AFZ	17.97	1	8	1.17	0.097
P05455	Lupus La protein	SSB	13.73	5	17	1.17	0.058
P61923	Coatomer subunit zeta-1	COPZ1	11.86	2	2	1.16	0.130
Q99538	Legumain	LGMN	4.39	1	4	1.15	0.038
Q9UKY7	Protein CDV3 homolog	CDV3	22.09	2	3	1.15	0.154
P14543	Nidogen-1	NID1	2.17	2	4	1.14	0.138
Q13347	Eukaryotic translation initiation factor 3 subunit I	EIF3I	12.00	3	6	1.14	0.096
P11940	Polyadenylate-binding protein 1	PABPC1	10.69	5	14	1.13	0.254
O43143	Putative pre-mRNA-splicing factor ATP-dependent RNA helicase DHX15	DHX15	5.03	3	7	1.13	0.071
Q07020	60S ribosomal protein L18	RPL18	27.13	4	19	1.13	0.249
P10909	Clusterin	CLU	23.39	8	60	1.13	0.058
P15880	40S ribosomal protein S2	RPS2	26.62	6	23	1.13	0.033
P21399	Cytoplasmic aconitate hydratase	ACO1	9.11	5	11	1.13	0.119
Q99536	Synaptic vesicle membrane protein VAT-1 homolog	VAT1	18.58	4	8	1.12	0.085
Q9UK76	Hematological and neurological expressed 1 protein	HN1	15.58	1	6	1.12	0.129
Q9P2E7	Protocadherin-10	PCDH10	1.25	1	2	1.12	0.133
Q96C90	Protein phosphatase 1 regulatory subunit 14B	PPP1R14B	8.84	1	1	1.12	0.054
P24534	Elongation factor 1-beta	EEF1B2	12.44	2	5	1.12	0.110
P35555	Fibrillin-1	FBN1	1.18	2	8	1.12	0.219
Q13867	Bleomycin hydrolase	BLMH	6.37	2	4	1.12	0.153
O75643	U5 small nuclear ribonucleoprotein 200 kDa helicase	SNRNP200	1.12	2	3	1.12	0.218
P52597	Heterogeneous nuclear ribonucleoprotein F	HNRNPF	14.46	2	21	1.12	0.086
P22061	Protein-L-isoaspartate(D-aspartate)	PCMT1	12.78	2	3	1.12	0.092
O95084	Serine protease 23	PRSS23	2.87	1	1	1.11	0.020
P62841	40S ribosomal protein S15	RPS15	26.90	2	5	1.11	0.086
Q7KZF4	Staphylococcal nuclease domain-containing protein 1	SND1	14.29	10	24	1.11	0.063
P45974	Ubiquitin carboxyl-terminal hydrolase 5	USP5	4.90	3	7	1.10	0.046

Q92688	Acidic leucine-rich nuclear phosphoprotein 32 family member B	ANP32B	13.94	1	29	1.10	0.151
P17612	cAMP-dependent protein kinase catalytic subunit alpha	PRKACA	3.42	1	3	1.10	0.138
Q15717	ELAV-like protein 1	ELAVL1	8.28	2	9	1.10	0.207
P01892	HLA class I histocompatibility antigen. A-2 alpha chain	HLA-A	13.42	3	15	1.10	0.057
Q14839	Chromodomain-helicase-DNA-binding protein 4	CHD4	0.63	1	2	1.10	0.071
O15067	Phosphoribosylformylglycinamidine synthase	PFAS	2.17	2	5	1.10	0.068
P14174	Macrophage migration inhibitory factor	MIF	9.57	1	2	1.10	0.182
Q14764	Major vault protein	MVP	10.53	6	14	1.10	0.086
O43809	Cleavage and polyadenylation specificity factor subunit 5	NUDT21	18.06	2	4	1.09	0.046
P31942	Heterogeneous nuclear ribonucleoprotein H3	HNRNPH3	4.91	1	3	1.09	0.042
Q07666	KH domain-containing. RNA-binding. signal transduction-associated protein 1	KHDRBS1	6.55	2	5	1.09	0.237
Q99426	Tubulin-folding cofactor B	TBCB	10.66	3	3	1.09	0.057
Q96KK5	Histone H2A type 1-H	HIST1H2AH	27.34	1	353	1.09	0.263
Q14195	Dihydropyrimidinase-related protein 3	DPYSL3	6.32	3	7	1.09	0.056
Q9Y4K0	Lysyl oxidase homolog 2	LOXL2	10.34	6	23	1.09	0.069
P61586	Transforming protein RhoA	RHOA	5.18	1	5	1.09	0.056
P35318	ADM	ADM	5.95	1	2	1.09	0.147
Q13201	Multimerin-1	MMRN1	28.26	25	201	1.09	0.040
Q15819	Ubiquitin-conjugating enzyme E2 variant 2	UBE2V2	11.72	2	5	1.09	0.136
P04632	Calpain small subunit 1	CAPNS1	25.00	4	17	1.08	0.037
P20645	Cation-dependent mannose-6-phosphate receptor	M6PR	10.11	2	3	1.08	0.155
Q10471	Polypeptide N-acetylgalactosaminyltransferase 2	GALNT2	6.48	3	6	1.08	0.120
Q02809	Procollagen-lysine.2-oxoglutarate 5-dioxygenase 1	PLOD1	12.10	7	11	1.08	0.094
P08253	72 kDa type IV collagenase	MMP2	21.06	10	51	1.08	0.128
Q13263	Transcription intermediary factor 1-beta	TRIM28	6.59	3	10	1.08	0.122
P61160	Actin-related protein 2	ACTR2	13.96	4	22	1.08	0.019
Q8IWU6	Extracellular sulfatase Sulf-1	SULF1	4.59	4	10	1.08	0.105
P63244	Guanine nucleotide-binding protein subunit beta-2-like 1	GNB2L1	11.99	3	5	1.08	0.189
P63000	Ras-related C3 botulinum toxin substrate 1	RAC1	7.81	2	3	1.08	0.264
P11387	DNA topoisomerase 1	TOP1	2.09	1	1	1.08	0.022
Q9UNN8	Endothelial protein C receptor	PROCR	11.34	2	9	1.08	0.086
Q9NS15	Latent-transforming growth factor beta-binding protein 3	LTBP3	1.30	1	4	1.08	0.032
P26640	Valyl-tRNA synthetase	VARS	4.27	3	6	1.08	0.086
P21810	Biglycan	BGN	25.82	7	131	1.08	0.031
P31937	3-hydroxyisobutyrate dehydrogenase. mitochondrial	HIBADH	4.17	1	2	1.07	0.087
P55209	Nucleosome assembly protein 1-like 1	NAP1L1	12.79	3	13	1.07	0.053
P52788	Spermine synthase	SMS	4.92	2	3	1.07	0.073
P62942	Peptidyl-prolyl cis-trans isomerase FKBP1A	FKBP1A	54.63	3	15	1.07	0.086
Q12841	Follistatin-related protein 1	FSTL1	11.69	4	12	1.07	0.129
Q99497	Protein DJ-1	PARK7	22.22	3	6	1.07	0.111
O94985	Calsyntenin-1	CLSTN1	6.32	5	7	1.07	0.130
O15511	Actin-related protein 2/3 complex subunit 5	ARPC5	12.58	1	4	1.07	0.107
Q13561	Dynactin subunit 2	DCTN2	12.47	5	8	1.07	0.095
Q9NUQ9	Protein FAM49B	FAM49B	5.86	1	4	1.07	0.054
P49411	Elongation factor Tu. mitochondrial	TUFM	15.49	6	12	1.07	0.034
P04264	Keratin. type II cytoskeletal 1	KRT1	5.43	3	5	1.07	0.178
P07384	Calpain-1 catalytic subunit	CAPN1	3.22	2	2	1.07	0.139
Q13283	Ras GTPase-activating protein-binding protein 1	G3BP1	17.17	5	13	1.07	0.072

O60220	Mitochondrial import inner membrane translocase subunit Tim8 A	TIMM8A	11.34	1	1	1.07	0.055
P23526	Adenosylhomocysteinase	AHCY	12.96	5	9	1.07	0.056
P03956	Interstitial collagenase	MMP1	5.76	2	5	1.07	0.077
P26583	High mobility group protein B2	HMGB2	12.92	3	13	1.07	0.067
P30153	Serine/threonine-protein phosphatase 2A 65 kDa regulatory subunit A alpha isoform	PPP2R1A	15.96	7	31	1.07	0.024
Q99798	Aconitate hydratase. mitochondrial	ACO2	5.13	3	7	1.07	0.128
Q7Z4V5	Hepatoma-derived growth factor-related protein 2	HDGFRP2	5.81	3	5	1.06	0.059
P20908	Collagen alpha-1(V) chain	COL5A1	0.98	2	2	1.06	0.041
Q9BPU6	Dihydropyrimidinase-related protein 5	DPYSL5	1.24	1	4	1.06	0.065
Q6WCQ1	Myosin phosphatase Rho-interacting protein	MPRIP	1.46	1	2	1.06	0.096
O43324	Eukaryotic translation elongation factor 1 epsilon-1	EEF1E1	14.37	1	2	1.06	0.061
Q9UDY2	Tight junction protein Z	TJP2	2.44	2	4	1.06	0.058
O14773	Tripeptidyl-peptidase 1	TPP1	9.77	3	5	1.06	0.144
P61221	ATP-binding cassette sub-family E member 1	ABCE1	1.84	1	1	1.06	0.137
P30041	Peroxiredoxin-6	PRDX6	26.34	4	14	1.06	0.072
Q99715	Collagen alpha-1(XII) chain	COL12A1	7.64	19	34	1.06	0.025
Q53GQ0	Estradiol 17-beta-dehydrogenase 12	HSD17B12	12.82	2	8	1.06	0.078
O14980	Exportin-1	XPO1	7.19	7	21	1.06	0.034
P06730	Eukaryotic translation initiation factor 4E	EIF4E	13.36	2	6	1.06	0.041
Q9UBS4	DnaJ homolog subfamily B member 11	DNAJB11	7.54	3	4	1.06	0.085
P19022	Cadherin-2	CDH2	1.43	2	5	1.06	0.127
P62888	60S ribosomal protein L30	RPL30	7.83	1	6	1.06	0.061
P33240	Cleavage stimulation factor subunit 2	CSTF2	3.64	1	4	1.06	0.102
P61163	Alpha-centractin	ACTR1A	12.23	3	10	1.06	0.123
P22004	Bone morphogenetic protein 6	BMP6	2.73	1	9	1.06	0.104
Q96AE4	Far upstream element-binding protein 1	FUBP1	5.43	3	9	1.06	0.084
O00232	26S proteasome non-ATPase regulatory subunit 12	PSMD12	7.46	2	5	1.06	0.062
P27695	DNA-(apurinic or apyrimidinic site) lyase	APEX1	15.09	4	13	1.06	0.065
P14866	Heterogeneous nuclear ribonucleoprotein L	HNRNPL	10.70	5	16	1.06	0.069
Q9BZZ5	Apoptosis inhibitor 5	API5	3.53	1	2	1.06	0.010
P26022	Pentraxin-related protein PTX3	PTX3	30.97	9	19	1.06	0.081
O75367	Core histone macro-H2A.1	H2AFY	5.38	1	4	1.05	0.137
P51452	Dual specificity protein phosphatase 3	DUSP3	6.49	1	1	1.05	0.141
O00299	Chloride intracellular channel protein 1	CLIC1	27.80	6	13	1.05	0.021
P24752	Acetyl-CoA acetyltransferase. mitochondrial	ACAT1	3.98	1	4	1.05	0.080
Q99613	Eukaryotic translation initiation factor 3 subunit C	EIF3C	6.90	4	11	1.05	0.073
Q96QV1	Hedgehog-interacting protein	HHIP	12.71	8	25	1.05	0.040
P58546	Myotrophin	MTPN	22.03	2	3	1.05	0.102
P49593	Protein phosphatase 1F	PPM1F	19.60	4	13	1.05	0.015
P19338	Nucleolin	NCL	9.44	7	12	1.05	0.032
P04899	Guanine nucleotide-binding protein G(i) subunit alpha-2	GNAI2	12.11	3	8	1.05	0.141
Q9NTK5	Obg-like ATPase 1	OLA1	3.79	1	9	1.05	0.021
Q6YHK3	CD109 antigen	CD109	8.93	8	21	1.05	0.020
P07996	Thrombospondin-1	THBS1	27.35	30	1627	1.05	0.077
P08962	CD63 antigen	CD63	7.56	2	2	1.05	0.092
P48147	Prolyl endopeptidase	PREP	4.23	3	4	1.05	0.045
P62195	26S protease regulatory subunit 8	PSMC5	10.10	2	16	1.05	0.016
P55058	Phospholipid transfer protein	PLTP	7.30	3	5	1.05	0.061
Q02952	A-kinase anchor protein 12	AKAP12	5.89	8	17	1.05	0.131
P33151	Cadherin-5	CDH5	14.54	9	44	1.05	0.076

P13010	ATP-dependent DNA helicase 2 subunit 2	XRCC5	25.14	11	28	1.05	0.017
O00391	Sulfhydryl oxidase 1	QSOX1	21.15	11	45	1.05	0.059
P17655	Calpain-2 catalytic subunit	CAPN2	8.29	4	13	1.05	0.085
Q16630	Cleavage and polyadenylation specificity factor subunit 6	CPSF6	4.54	1	5	1.05	0.083
P55884	Eukaryotic translation initiation factor 3 subunit B	EIF3B	7.99	5	13	1.05	0.028
P25786	Proteasome subunit alpha type-1	PSMA1	19.39	6	16	1.05	0.065
Q13151	Heterogeneous nuclear ribonucleoprotein A0	HNRNPA0	6.56	1	4	1.05	0.077
P98160	Basement membrane-specific heparan sulfate proteoglycan core protein	HSPG2	24.46	67	1085	1.05	0.025
P05067	Amyloid beta A4 protein	APP	14.29	9	57	1.05	0.083
P50395	Rab GDP dissociation inhibitor beta	GDI2	28.76	9	40	1.05	0.028

*Proteins whose expression is increased (≥5%) in HUVECs after treatment with sEng are listed. From left to right, the different columns indicate: Protein code (UniProt); Protein name; Gene name; Coverage, percentage of the protein sequence covered by identified peptides; Number of unique peptide sequences identified; PSMs, "peptide spectrum matches" or total number of peptides identified corresponding to the specific protein; Average fold-induction of each protein after treatment with sEng compared to control; and SD, standard deviation. Those proteins marked in red are considered statistically significant with p-value <0.05.

Supplementary Table S2. Downregulated proteins upon sEng treatment (≥5%)*

Accession	Protein Name	Gene Name	ΣCoverage	ΣUnique peptides	ΣPSMs	solEng/Control Average	SD
A6NIZ1	Ras-related protein Rap-1b-like protein	RAP1BL	11.96	2	3	0.95	0.013
Q8N392	Rho GTPase-activating protein 18	ARHGAP18	1.66	1	2	0.95	0.063
P31948	Stress-induced-phosphoprotein 1	STIP1	10.68	6	15	0.95	0.068
Q15366	Poly(rC)-binding protein 2	PCBP2	24.11	4	13	0.95	0.091
O00571	ATP-dependent RNA helicase DDX3X	DDX3X	8.46	4	12	0.95	0.031
P84103	Splicing factor. arginine/serine-rich 3	SRSF3	9.76	2	3	0.95	0.032
P23381	Tryptophanyl-tRNA synthetase. cytoplasmic	WARS	16.35	5	16	0.95	0.025
P62328	Thymosin beta-4	TMSB4X	15.91	1	6	0.95	0.112
Q9UBR2	Cathepsin Z	CTSZ	7.26	2	4	0.95	0.039
P50991	T-complex protein 1 subunit delta	CCT4	12.62	4	22	0.95	0.049
P08758	Annexin A5	ANXA5	55.63	13	55	0.95	0.064
O75131	Copine-3	CPNE3	2.98	1	2	0.95	0.079
P28070	Proteasome subunit beta type-4	PSMB4	9.47	2	3	0.95	0.103
Q8IYB3	Serine/arginine repetitive matrix protein 1	SRRM1	2.77	1	1	0.95	0.107
P61916	Epididymal secretory protein E1	NPC2	4.64	1	1	0.95	0.056
P06753	Tropomyosin alpha-3 chain-isoform 2	TPM3	17.04	2	26	0.95	0.047
P67936	Tropomyosin alpha-4 chain	TPM4	37.10	8	43	0.95	0.062
O75947	ATP synthase subunit d. mitochondrial	ATP5H	14.29	2	3	0.95	0.046
P31946	14-3-3 protein beta/alpha	YWHAB	48.37	5	149	0.95	0.021
P62851	40S ribosomal protein S25	RPS25	20.80	3	7	0.95	0.092
P30050	60S ribosomal protein L12	RPL12	24.24	3	4	0.95	0.123
P36578	60S ribosomal protein L4	RPL4	23.42	8	37	0.95	0.031
P05388	60S acidic ribosomal protein P0	RPLP0	19.56	5	29	0.95	0.083
P18669	Phosphoglycerate mutase 1	PGAM1	40.16	7	23	0.95	0.024
P00387	NADH-cytochrome b5 reductase 3	CYB5R3	25.58	5	13	0.95	0.055
P04080	Cystatin-B	CSTB	33.67	2	4	0.95	0.053
P08195	4F2 cell-surface antigen heavy chain	SLC3A2	1.90	1	1	0.95	0.072
P38646	Stress-70 protein. mitochondrial	HSPA9	15.76	10	28	0.95	0.034
Q8NBS9	Thioredoxin domain-containing protein 5	TXNDC5	33.56	12	154	0.95	0.020
P08729	Keratin. type II cytoskeletal 7	KRT7	25.59	11	31	0.95	0.028
P26373	60S ribosomal protein L13	RPL13	18.96	4	11	0.95	0.035
P08670	Vimentin	VIM	56.01	26	495	0.95	0.087
P52565	Rho GDP-dissociation inhibitor 1	ARHGDIA	24.02	6	19	0.95	0.086
P16949	Stathmin	STMN1	34.90	5	25	0.95	0.151
P27824	Calnexin	CANX	28.89	15	52	0.95	0.037
P62266	40S ribosomal protein S23	RPS23	15.38	2	10	0.95	0.029
Q7Z6Z7	E3 ubiquitin-protein ligase HUWE1	HUWE1	0.32	1	1	0.95	0.060
P83731	60S ribosomal protein L24	RPL24	18.47	3	8	0.94	0.015
Q08257	Quinone oxidoreductase	CRYZ	16.41	3	12	0.94	0.060
P52272	Heterogeneous nuclear ribonucleoprotein M	HNRNPM	15.21	7	16	0.94	0.016
P40926	Malate dehydrogenase. mitochondrial	MDH2	23.67	6	31	0.94	0.056
P05141	ADP/ATP translocase 2	SLC25A5	18.79	5	19	0.94	0.075
Q9BS40	Latexin	LXN	13.51	2	11	0.94	0.016
P11413	Glucose-6-phosphate 1-dehydrogenase	G6PD	12.43	6	9	0.94	0.076
P50914	60S ribosomal protein L14	RPL14	17.67	4	10	0.94	0.061
P25398	40S ribosomal protein S12	RPS12	13.64	2	20	0.94	0.099
Q99623	Prohibitin-2	PHB2	19.40	4	9	0.94	0.079

P05387	60S acidic ribosomal protein P2	RPLP2	66.96	4	51	0.94	0.108
O00410	Importin-5	IPO5	8.48	7	10	0.94	0.058
P17812	CTP synthase 1	CTPS1	4.06	2	6	0.94	0.026
O60869	Endothelial differentiation-related factor 1	EDF1	22.97	3	9	0.94	0.029
P26196	Probable ATP-dependent RNA helicase DDX6	DDX6	4.97	1	4	0.94	0.217
O60814	Histone H2B type 1-K	HIST1H2BK	47.62	2	267	0.94	0.051
P34897	Serine hydroxymethyltransferase. mitochondrial	SHMT2	11.51	3	7	0.94	0.067
P11279	Lysosome-associated membrane glycoprotein 1	LAMP1	4.80	2	3	0.94	0.101
Q9Y265	RuvB-like 1	RUVBL1	5.70	2	5	0.94	0.051
Q00325	Phosphate carrier protein. mitochondrial	SLC25A3	7.18	2	8	0.94	0.089
P62424	60S ribosomal protein L7a	RPL7A	15.79	4	7	0.94	0.050
P61978	Heterogeneous nuclear ribonucleoprotein K	HNRNPK	23.97	9	65	0.94	0.043
P23284	Peptidyl-prolyl cis-trans isomerase B	PPIB	29.17	6	23	0.94	0.075
P06733	Alpha-enolase	ENO1	44.70	18	164	0.94	0.056
P62491	Ras-related protein Rab-11A	RAB11A	7.41	2	7	0.93	0.053
Q15293	Reticulocalbin-1	RCN1	25.98	6	12	0.93	0.029
P48047	ATP synthase subunit	ATP5O	11.27	2	8	0.93	0.076
P61353	60S ribosomal protein L27	RPL27	14.71	2	8	0.93	0.010
P48643	T-complex protein 1 subunit epsilon	CCT5	14.42	7	16	0.93	0.068
O75608	Acyl-protein thioesterase 1	LYPLA1	4.78	1	6	0.93	0.058
Q9BRX8	Uncharacterized protein C10orf58	FAM213A	24.45	5	19	0.93	0.030
Q96HC4	PDZ and LIM domain protein 5	PDLIM5	4.53	2	6	0.93	0.047
P60174	Triosephosphate isomerase	TPI1	44.98	8	53	0.93	0.064
Q9P0L0	Vesicle-associated membrane protein-associated protein A	VAPA	6.83	2	9	0.93	0.067
P63241	Eukaryotic translation initiation factor 5A-1	EIF5A	27.92	3	21	0.93	0.035
Q07065	Cytoskeleton-associated protein 4	CKAP4	29.57	13	34	0.93	0.070
P51991	Heterogeneous nuclear ribonucleoprotein A3	HNRNPA3	20.37	5	9	0.93	0.064
Q9Y512	Sorting and assembly machinery component 50 homolog	SAMM50	2.13	1	1	0.93	0.048
O15145	Actin-related protein 2/3 complex subunit 3	ARPC3	11.24	2	5	0.92	0.012
P62241	40S ribosomal protein S8	RPS8	21.15	4	15	0.92	0.049
Q02790	Peptidyl-prolyl cis-trans isomerase FKBP4	FKBP4	7.84	3	7	0.92	0.165
P22695	Cytochrome b-c1 complex subunit 2. mitochondrial	UQCRC2	9.49	3	11	0.92	0.084
Q92522	Histone H1x	H1FX	7.04	1	4	0.92	0.090
Q13045	Protein flightless-1 homolog	FLII	1.10	1	4	0.92	0.030
P84090	Enhancer of rudimentary homolog	ERH	5.77	1	2	0.92	0.038
P62805	Histone H4	HIST1H4A	57.28	9	224	0.92	0.090
P14625	Endoplasmin	HSP90B1	30.39	20	105	0.92	0.057
P61604	10 kDa heat shock protein. mitochondrial	HSPE1	21.57	2	3	0.92	0.077
P60900	Proteasome subunit alpha type-6	PSMA6	25.20	5	11	0.92	0.018
P62312	U6 snRNA-associated Sm-like protein LSm6	LSM6	13.75	1	1	0.92	0.032
P16402	Histone H1.3	HIST1H1D	18.55	3	20	0.92	0.052
P68431	Histone H3.1	HIST1H3A	14.71	3	8	0.92	0.067
P01033	Metalloproteinase inhibitor 1	TIMP1	5.80	1	1	0.92	0.098
P46783	40S ribosomal protein S10	RPS10	32.12	5	12	0.91	0.012
P69905	Hemoglobin subunit alpha	HBA1	28.17	3	8	0.91	0.230
P35659	Protein DEK	DEK	3.47	1	2	0.91	0.089
P17096	High mobility group protein HMG-I/HMG-Y	HMGA1	23.36	3	64	0.91	0.165
P61619	Protein transport protein Sec61 subunit alpha isoform 1	SEC61A1	1.89	1	4	0.91	0.087
O43852	Calumenin	CALU	24.44	6	16	0.91	0.058
P62750	60S ribosomal protein L23a	RPL23A	26.92	4	6	0.91	0.035

P38159	Heterogeneous nuclear ribonucleoprotein G	RBMX	6.65	2	6	0.91	0.051
P42766	60S ribosomal protein L35	RPL35	8.13	1	1	0.91	0.037
Q14203	Dynactin subunit 1	DCTN1	3.83	3	5	0.91	0.084
P09972	Fructose-bisphosphate aldolase C	ALDOC	6.32	1	4	0.90	0.061
P62277	40S ribosomal protein S13	RPS13	21.19	3	10	0.90	0.079
P62158	Calmodulin	CALM1	16.11	3	7	0.89	0.123
P52907	F-actin-capping protein subunit alpha-1	CAPZA1	16.08	3	4	0.89	0.059
Q96PK6	RNA-binding protein 14	RBM14	6.13	3	7	0.89	0.049
P62269	40S ribosomal protein S18	RPS18	21.05	3	5	0.89	0.098
Q8NI22	Multiple coagulation factor deficiency protein 2	MCFD2	16.44	1	2	0.89	0.083
P49721	Proteasome subunit beta type-2	PSMB2	14.43	3	6	0.89	0.113
P49207	60S ribosomal protein L34	RPL34	5.98	1	2	0.88	0.061
Q8WW12	PEST proteolytic signal-containing nuclear protein	PCNP	15.17	2	5	0.88	0.163
P21796	Voltage-dependent anion-selective channel protein 1	VDAC1	19.43	4	8	0.87	0.013
Q15363	Transmembrane emp24 domain-containing protein 2	TMED2	24.38	4	22	0.87	0.089
P30408	Transmembrane 4 L6 family member 1	TM4SF1	4.46	1	3	0.87	0.161
O75964	ATP synthase subunit g. mitochondrial	ATP5L	10.68	1	2	0.86	0.085
O43488	Aflatoxin B1 aldehyde reductase member 2	AKR7A2	9.47	2	4	0.86	0.087
P13645	Keratin. type I cytoskeletal 10	KRT10	7.71	3	5	0.86	0.181
P47914	60S ribosomal protein L29	RPL29	9.43	1	7	0.85	0.005
P62820	Ras-related protein Rab-1A	RAB1A	13.66	1	6	0.85	0.218
P24539	ATP synthase subunit b. mitochondrial	ATP5F1	10.94	3	12	0.84	0.126
P99999	Cytochrome c	CYCS	30.48	3	14	0.83	0.003
P28066	Proteasome subunit alpha type-5	PSMA5	17.84	4	14	0.80	0.107
P05114	Non-histone chromosomal protein HMG-14	HMGN1	23.00	2	5	0.78	0.042

*Proteins whose expression is decreased (≥5%) in HUVECs after treatment with sEng are listed. From left to right, the different columns indicate: Protein code (UniProt); Protein name; Gene name; Coverage, percentage of the protein sequence covered by identified peptides; Number of unique peptide sequences identified; PSMs, "peptide spectrum matches" or total number of peptides identified corresponding to the specific protein; Average fold-induction of each protein after treatment with sEng compared to control; and SD, standard deviation. Those proteins marked in green are considered statistically significant with p-value <0.05.
